# T. S. M. A. Correction

**Published:** 1887-09

**Authors:** 


### Society Notes.

### T. S. M. A.

Dr. M. K. Lott, of Belton, Texas, claims that there are certain inaccuracies in the Secretary's Minutes of the late meeting of the Texas State Medical Association, as published in the Transactions just issued, and points them out in a letter to us. We make the following extract from his letter, in justice to him, and to all concerned. There was considerable confusion on the floor at the time, and we think the Secretary is excusable if he did get the names of "movers" a little mixed, so many were speaking or "moving" at one time.

"I note some inaccuracies in the Proceedings; for instance: on page 67. "Dr. Lott rose to a point of personal privelege" &c. "Had attempted every ligitimate means in his power to that end, *and that the profession of Belton would not settle it.*" [Lott's italics.] The words underscored, I did not use; but I said that "Dr. Ghent would not agree for the profession of Belton (who were willing) to settle it. I proposed to pick a physician from the county and [Dr.] Ghent to pick one, they to select a third, and we were to abide their verdict, whatever it was. This, [Dr.] Ghent refused; when I resorted to the only means left me, &c.

Again: on same page, you say, "Dr. Lott challenged Dr. Allen's vote as representing Bell county." This I did not do: I said not a word about it; nor do I understand that his vote was challenged in my interest, but in the interest of truth and right. The Secretary of Bell County Medical Association, [Dr. Tally], was on the floor, and challenged the vote, he being aware that the Bell County Medical Association had said to her delegates that she would not be represented, and that Dr. Allen knew it, being President of the Bell County Medical Association and presiding at the time. Just how "Dr. Allen withdrew his vote for the sake of harmony," when he had no vote to withdraw, is not well comprehended."

"These points affect me vitally and I think the correction as I have here made it, should appear in your next Journal."

[Signed] M. K. Lott.

We give place to the above, with pleasure, but we do not attach the importance to the question as to who challenged Dr. Allen's vote, that Dr. Lott does ;—it *was* challenged, and by a member of the Bell County Medical Association, and we thought at the time, and so recorded it, that it was challenged by Dr. Lott, and it is immaterial whether it was Dr. Lott or Dr. Tally.

